# Expanding the Scope of Molecular Glues: TRIM21 as a Multimeric Protein Degrader

**DOI:** 10.1002/mco2.70178

**Published:** 2025-04-14

**Authors:** Kai Huang, Anqi Zhou, Xiangxiang Zhou

**Affiliations:** ^1^ Department of Medical Oncology Qilu Hospital of Shandong University Jinan China; ^2^ Department of Hematology Shandong Provincial Hospital Affiliated to Shandong First Medical University Jinan Shandong China

1

In a recent study published on *Cell*, Lu et al. [1] introduce a novel targeted protein degradation (TPD) strategy that employs Tripartite motif‐containing protein 21 (TRIM21), an E3 ligase activated through clustering, to achieve multimeric protein degradation with remarkable selectivity.

The field of TPD has expanded rapidly with the development of molecular glues (MGs) and proteolysis‐targeting chimeras (PROTACs), which use E3 ubiquitin ligases to tag proteins for degradation [2]. However, current TPD methods rely heavily on E3 ligases such as Cereblon and Von Hippel–Lindau tumor suppressor, which are broadly expressed and lack conditional activity, posing challenges for selective targeting, especially in complex protein assemblies [3].

An additional challenge lies in selectively targeting multimeric proteins, which are often implicated in disease due to their aggregated forms, as seen in neurodegenerative disorders and certain cancers. Current TPD strategies typically lack the ability to distinguish between monomeric and multimeric forms of a protein, resulting in potential off‐target effects and limited efficacy for diseases marked by aberrant multimerization. These limitations have highlighted the need for degraders that can selectively target multimeric assemblies with minimal impact on their monomeric counterparts, which may retain essential cellular functions.

The study focuses on (S)‐ACE‐OH, a metabolite derived from the antipsychotic acepromazine, which acts as a MG facilitating interaction between TRIM21 and the nuclear pore protein NUP98, leading to selective degradation of multimeric protein structures within the nuclear pore complex (NPC) (Figure 1). Specifically, the authors demonstrate that treatment with (S)‐ACE‐OH leads to the depletion of NPC subunits, as evidenced by proteomics analyses and transmission electron microscopy, which reveal structural disintegration of the inner NPC ring.

**FIGURE 1:**
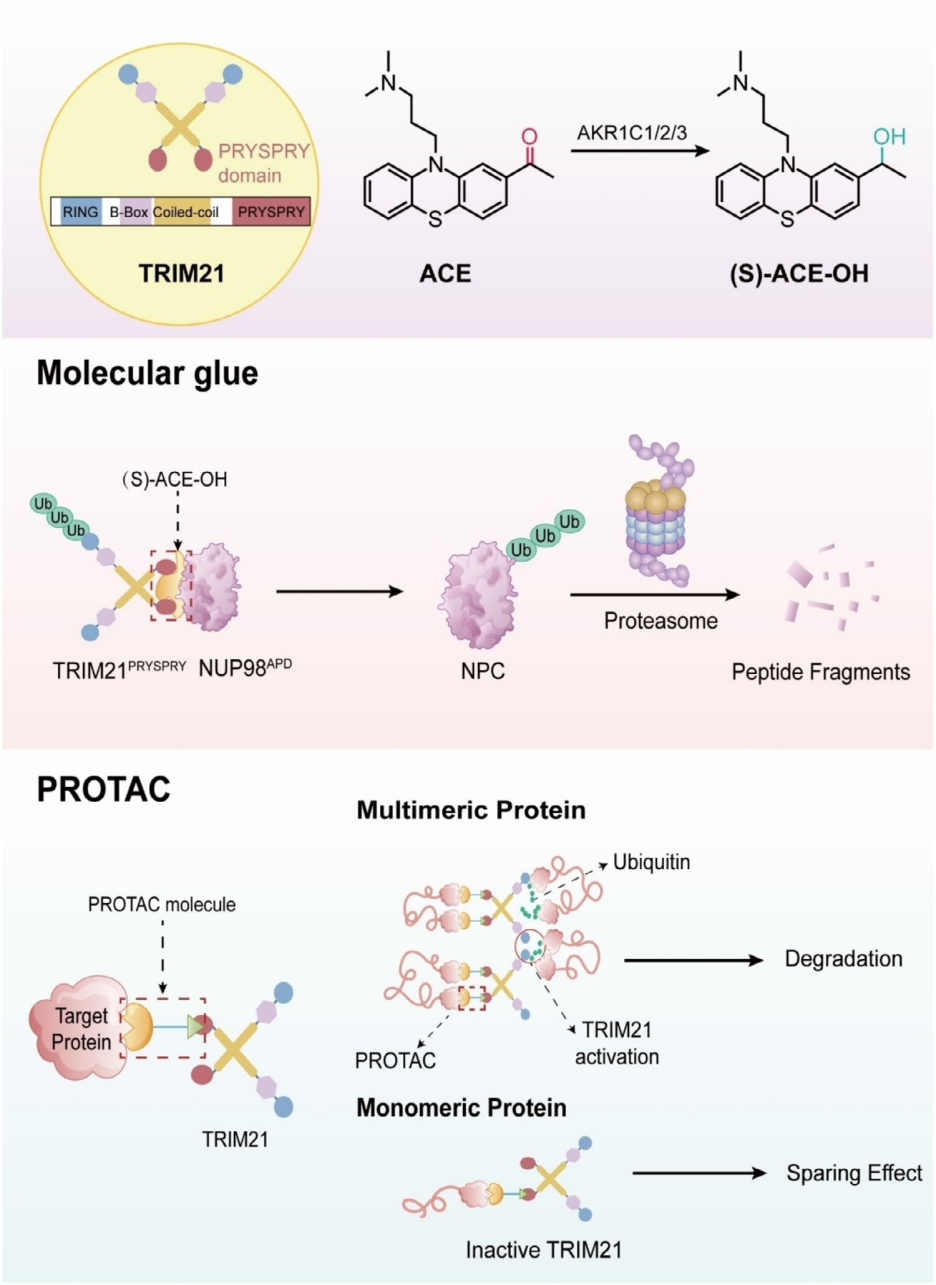
Strategy of TRIM21‐based molecular glue and PROTAC degraders. (S)‐ACE‐OH, a metabolite derived from the antipsychotic drug acetophenazine, functions as a molecular glue degrader of the nuclear pore complex. It facilitates the interaction between the PRYSPRY domain of the E3 ligase TRIM21 and the nucleoporin NUP98, leading to proteasomal degradation of nucleoporins within the NPC. The activation of TRIM21's E3 ubiquitin ligase activity relies on its polymeric protein substrates inducing TRIM21 aggregation. A PROTAC designed to target the unique ligandable pocket within the PRYSPRY domain of TRIM21 enables selective degradation of protein aggregates without affecting the biological functions of monomeric proteins. This strategy offers a potential approach for addressing pathological protein aggregation in diseases. ACE, acetylpromazine; AKR1C1/2/3, aldo‐keto reductase family 1 member C1/2/3; NUP98, nuclear pore complex protein 98; NPC, nuclear pore complex; PROTAC, proteolysis‐targeting chimera; TRIM21, tri‐partite motif‐containing 21.

The researchers first identify (S)‐ACE‐OH as a MG that mediates an interaction between TRIM21 and NUP98, a protein critical to the integrity of the NPC. Using CRISPR‐based screening, the authors confirmed TRIM21 as essential for the cytotoxic effects of (S)‐ACE‐OH, particularly under interferon‐gamma conditions, which induce TRIM21 expression. Through a competitive cell growth assay in interferon‐gamma‐stimulated cancer cell lines, they observed that (S)‐ACE‐OH selectively targets multimeric NUP98‐containing complexes, reducing cell viability by degrading the NPC's inner ring. Proteomic quantification demonstrated a significant reduction in NUP98 levels, underscoring the compound's potency. The work highlights how multimeric proteins, but not monomeric counterparts, induce the clustering of TRIM21, a mechanism necessary for activating TRIM21's E3 ligase function. This clustering‐dependent activation serves as a unique mechanism for selectively degrading multimeric protein assemblies within the cell. Importantly, this approach offers a distinct advantage over previous TPD methods that primarily rely on monovalent binding mechanisms, which struggle to distinguish between monomeric and multimeric protein states.

A pivotal aspect of the study involved structural analyses of the TRIM21–(S)‐ACE‐OH complex. The researchers resolved cocrystal structures that reveal a key binding pocket within TRIM21's PRYSPRY domain, where (S)‐ACE‐OH binds to facilitate TRIM21–NUP98 interaction. Structural comparisons further revealed that the configuration of (S)‐ACE‐OH within this binding pocket is critical for complex formation with NUP98, as (R)‐ACE‐OH and acepromazine, though structurally similar, fail to engage TRIM21 similarly. This structural insight not only confirms the specificity of (S)‐ACE‐OH but also provides a foundation for engineering next‐generation TRIM21‐based degraders with enhanced selectivity for disease‐relevant multimeric proteins.

The study then investigates the downstream effects of NPC degradation. By targeting NUP98 within the NPC, (S)‐ACE‐OH induces the degradation of specific NPC subunits, disrupting nucleocytoplasmic trafficking and leading to characteristic cellular changes. High‐resolution microscopy and proteomics revealed that degradation initially impacts the NPC's inner ring, including proteins such as NUP35 and NUP155, while the outer ring and nuclear basket remain relatively intact. This selective degradation was confirmed through Western blot analysis and transmission electron microscopy, both showing a gradual collapse of the NPC architecture as TRIM21 initiates the degradation process. The authors suggest that TRIM21's modular structure, which positions its ubiquitin‐binding RING domain at a distance from the PRYSPRY domain, enables it to ubiquitinate nearby proteins within the NPC, thus selectively dismantling the multimeric assembly.

Finally, the authors examined how mutations in NUP98 confer resistance to (S)‐ACE‐OH. Using CRISPR‐suppressor scanning, they identified key residues within NUP98's autoproteolytic domain essential for (S)‐ACE‐OH‐induced degradation. Mutations disrupting this domain impair TRIM21 recruitment to the NPC, effectively blocking (S)‐ACE‐OH's degradative action. To further probe the interaction between (S)‐ACE‐OH, TRIM21, and NUP98, the authors engineered an in vitro condensate assay that simulates multimerized interactions within the NPC. They found that (S)‐ACE‐OH specifically enriches TRIM21 in these condensates, confirming that clustering of multimeric structures is necessary for TRIM21 activation. This clustering effect, coupled with structural insights into (S)‐ACE‐OH's binding mode, underlines the potential of this MG to achieve precise and selective degradation of disease‐relevant protein assemblies.

By showcasing a TRIM21‐based degrader selective for multimeric proteins, the authors provide a new path for targeting complex protein assemblies in diseases like cancer and neurodegeneration. This clustering‐dependent TRIM21 activation offers a precision that could be expanded to explore other E3 ligases that activate under specific structural conditions, broadening the therapeutic landscape for TPD. The unique ligandable pocket within the PRYSPRY domain opens possibilities for rational design of TRIM21‐based degraders that might selectively regulate protein complexes in diverse cellular environments. Beyond the NPC, this system could potentially address pathological protein aggregations in diseases such as Alzheimer's, suggesting that targeted TPD could offer a novel approach to manage such conditions. Future work could involve systematic screening of MGs that enhance TRIM21 clustering or the development of engineered TRIM21 variants with optimized E3 ligase activity for specific multimeric substrates.

Recent preprints further expand our understanding of TRIM21‐based MGs [4, 5]. Cheng et al. [5] demonstrate that the TRIM21–NUP98 interface can accommodate structurally diverse MGs, such as PRLX‐93936 and BMS‐214662, reinforcing the therapeutic potential of TRIM21 as an E3 ligase for targeting multimeric proteins. Meanwhile, Scemama de Gialluly et al. [4] identify PRLX‐93936 and BMS‐214662 as potent MGs that leverage TRIM21 to degrade nucleoporins, revealing their high cellular potency and potential clinical applications. These findings further highlight the versatility of TRIM21 in mediating targeted degradation beyond the originally identified (S)‐ACE‐OH compound.

This study also raises compelling questions about the limits of selective degradation—such as achieving context‐specific activity without off‐target effects—and invites further engineering of TRIM21 or similar ligases for broader applications. By advancing our ability to target multimeric proteins, Lu et al. [1] provide a conceptual shift in TPD, paving the way for new therapies that tackle the molecular foundations of complex diseases.

## Author Contributions

K. H. wrote the draft of the manuscript. A. Z. made the figure. X. Z. initiated the idea and edited the manuscript. All authors have read and approved the final manuscript.

## Conflicts of Interest

The authors declare no conflicts of interest.

2

## Ethics Statement

The authors have nothing to report.

## Data Availability

The authors have nothing to report.
